# Massive underrepresentation of Arabs in genomic studies of common disease

**DOI:** 10.1186/s13073-023-01254-8

**Published:** 2023-11-22

**Authors:** Romit Bhattacharya, NingNing Chen, Injeong Shim, Hiroyuki Kuwahara, Xin Gao, Fowzan S. Alkuraya, Akl C. Fahed

**Affiliations:** 1 Department of Medicine, Cardiovascular Research Center, Massachusetts General Hospital, Harvard Medical School, 185 Cambridge Street | CPZN 3.128, Boston, MA 02114 USA; 2 Department of Medicine, Center for Genomic Medicine, Massachusetts General Hospital, Harvard Medical School, Boston, MA USA; 3 Cardiovascular Disease Initiative, Broad Institute of MIT and Harvard, Cambridge, MA USA; 4 Computational Biosciences Research Center (CBRC), King Abdullah University of Science and Technology (KAUST), Thuwal, Kingdom of Saudi Arabia; 5 Computer Science Program, Computer, Electrical and Mathematical Sciences and Engineering Division, King Abdullah University of Science and Technology (KAUST), Thuwal, Kingdom of Saudi Arabia; 6 Department of Digital Health, Samsung Medical Center, Samsung Advanced Institute for Health Sciences & Technology, Sungkyunkwan University, Seoul, Republic of South Korea; 7 Department of Translational Genomics, Center for Genomic Medicine, King Faisal Specialist Hospital and Research Center, Riyadh, Kingdom of Saudi Arabia

**Keywords:** Arab, Middle East, Underrepresentation, Diversity, Genomic, Polygenic score, GWAS

## Abstract

Arabs represent 5% of the world population and have a high prevalence of common disease, yet remain greatly underrepresented in genome-wide association studies, where only 1 in 600 individuals are Arab. We highlight the persistent and unaddressed underrepresentation of Arabs in genomic databases and discuss its impact on public health genomics and missed opportunities for biological discovery.

## Arabs, common complex disease, and healthcare disparities

Arab people represent about 5% of the world population and are an ancestrally diverse group with unique health considerations. Arabs can be defined by their geographic histories, religious practices, ethnicities, and countries of origin. The countries of the Arab world span the Arabian Peninsula, the Levant, and North Africa and are very diverse economically — including some of the wealthiest and poorest countries in the world — and genetically through extensive evidence of admixture found among present-day Arabs. However, while Arabs are ancestrally diverse, they have (1) genetically distinct signatures [1] and (2) unique risk factors for common diseases. These include waterpipe smoking — a key driver for risk of heart disease and cancer, some of the highest rates of vitamin D deficiency worldwide — attributed to dark skin and conservative clothing [2] and consanguinity leading to autozygosity.

Arabs are also present in diaspora, and as such, the risk of propagating healthcare disparities is a problem for not only the Arab world but also the West. Several Arab countries have witnessed large waves of immigration to the West due to conflict and political or economic breakdown. In the US alone, it is estimated that there are 3.7 million Arabs although it is likely that actual estimates are larger due to underreporting. Limited studies on Arab Americans observed a higher age-adjusted mortality rate than non-Arab Americans raising concerns for possible disparities in those populations [3]. With extensive European, Asian, and African admixture, Arabs can present as White, or non-White which allows misidentification in Western datasets and confounds attempts at disaggregation of data. In fact, even the US Census does not acknowledge Arabs as a separate category and instead sees them as “White.”

There is a rising epidemic of complex diseases in countries of the Arab world. The increasing rates of cardiovascular disease, diabetes, dyslipidemia, and cancer are often ascribed to environmental, behavioral, or economic factors. For decades, communicable diseases in low- and middle-income countries of the Arab world were the public health priority, but today nearly 80% of deaths are attributable to non-communicable diseases [4]. Common diseases are highly heritable and it remains unclear how genomic background may drive risk among Arabs for common diseases where there is a large public health burden. The interplay of genomic and nongenomic risk for many common diseases is increasingly appreciated in European-ancestry datasets and, more recently, other world populations with growing genomic and clinical datasets. For example, recent insights from individuals of South Asian ancestry identified increased risk for cardiovascular disease not captured by known conventional risk factors [5]. Whether this is true for Arabs is unknown.

## Massive and persistent underrepresentation of Arabs in genome-wide association studies

Genomic databases globally have suffered from limited ancestral diversity, and Martin et al. highlighted how this could propagate health disparities through predictable overrepresentation of genetic minorities — demonstrating that in 2019 despite only 16% of the global population being of European descent, ~ 79% of all genome-wide association studies (GWAS) participants were of European descent [6]. Arab populations are among the populations least represented in GWAS and large population-based biobanks, along with peoples of Oceania, Southeast Asia, Native American nations, and parts of Africa. Given high levels of endogamy and consanguinity in the Arab population, the inclusion of Arabs in such datasets has been an area of interest, but has not been a focus globally. Currently, in the GWAS Catalog, 88% of individuals are of European ancestry and 5.8% are East Asian (Biobank Japan and China Kadoorie Biobank being among the largest contributors from East Asia). By contrast, the relative inclusion of other ancestry groups has consistently languished at < 1.6%, with Hispanic, South Asian, and Greater Middle Eastern populations making up < 1% of the participants in published GWAS studies.

**Fig. 1:**
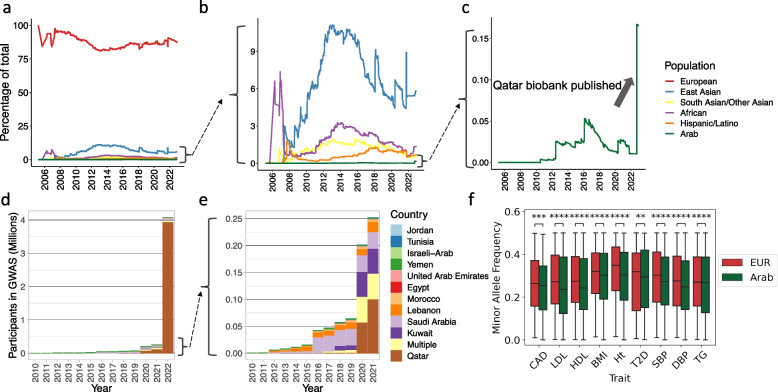
Proportional representation of Arabs in genomic studies of common disease. **a** Proportional representation of published GWAS study participants between 2006 and 2022 for individuals of 6 ancestral groups. The *y*-axis represents the cumulative percentage of the total. **b** Zoomed-in plot of **a** which excludes European-ancestry participants highlighting increase in representation of East Asian, South Asian, African, and Hispanic/Latino-ancestry group participants over time, but a persistent representation near zero for Arabs. **c** Zoomed-in plot of **b** which shows only Arab participants highlighting the recent increase in representation due to the publication of data from the Qatar Biobank (QBB). **d** Participants in published GWAS studies by the Arab country of origin between 2010 and 2022. By 2022, 96.2% of the Arab participants are from Qatar and the remaining 3.8% are from 10 other countries. **e** Zoomed-in plot of **d** which excludes the data of 2022 to highlight the breakdown of participants by the remaining countries. **f** For each of 9 cardiometabolic traits, we compare the minor allele frequency (MAF) of causal variants associated with the trait among individuals of European ancestry in the UK Biobank (*N* = 361,194) to their frequency in an indigenous Arab cohort from Saudi Arabia (*N* = 1017). Causal variants were obtained from fine mapping results performed using FINEMAP v1.3.1 and SuSiE v0.8.1.0521, available from the Funcane Lab (www.finucanelab.org/data). Across all 9 traits, the MAF of causal variants was significantly lower in Indigenous Arabs compared to European-ancestry individuals. CAD coronary artery disease, LDL low-density lipoprotein cholesterol, HDL high-density lipoprotein cholesterol, BMI body mass index, Ht height, T2D type 2 diabetes mellitus, SBP systolic blood pressure, DBP diastolic blood pressure, TG triglyceride. Mann–Whitney *U* test was used for comparison. “****” denotes *p* < 1.00e − 04, “***” denotes 1.00e − 04 < *p* ≤ 1.00e − 03, and “**” denotes 1.00e − 03 < *p* ≤ 1.00e − 02

We used the GWAS Catalog (https://www.ebi.ac.uk/gwas/) to quantify the representation trend for Arab individuals over 18 years — on aggregate and broken down by country — and find extraordinary underrepresentation in GWAS studies. When we examined Arab populations specifically defined by country of origin (in contrast to the grouping variable “Greater Middle Eastern” that is often used), we found approximately 0.17% of the GWAS Catalog was Arab — meaning about 1 in 600 individuals in published GWAS studies are currently of Arab ancestry (compared to 523 in 600 are of European ancestry). What is more discouraging is that this disparity has not been sufficiently addressed over time (Fig. 1a). When considering only non-European genomes, Arabs are noted to be consistently near 0% since the start of the GWAS Catalog (Fig. 1b). In fact, representation from the majority of countries considered to comprise the Arab world is lacking (Fig. 1d, e). Genomes from Algeria, Iraq, Libya, Oman, Somalia, and Syria are all completely absent.

The Qatar Biobank (QBB) has published data on ~ 15,000 individuals, and this represents the single largest contribution to Arab genomic representation to date (Fig. 1c) [7]. With even this modest addition in number, the contribution to genomics has been large with the initial GWAS from the QBB identifying 24.6 million previously unknown variants, and numerous novel disease-associated variants predominantly from 5 newly identified non-admixed subclusters within the Qatari population [7, 8]. The addition of QBB data to the GWAS Catalog represents a great advance, but additionally highlights the necessity and urgency of increasing representation from other Arab countries (Fig. 1d, e). While the contributions of the QBB are commendable, Qatar is not representative of all Arabs, for instance the distant North African or Levantine countries, as genetic ancestry varies greatly across the region.

## Missed opportunities in disease prevention and discovery

Discoveries from GWAS have begun to move into the clinical space through implementation of polygenic risk scores (PRS). It is now well-established that lack of diversity in reference genomes and published GWAS limit portability and accuracy of PRS prediction in non-European ancestries. The issues begin with SNP array imputation where reference panels are predominantly of European origin, and thus, when linkage disequilibrium (LD) blocks differ across ancestries, variation in diverse populations can be completely obscured ^6^. Differences in minor allele frequency (MAF) and effect size of variants among individuals of different genetic ancestries also lead to reduced accuracy of prediction — and increase the likelihood of propagating healthcare disparities ^6^. When comparing the MAF of causal variants associated with 9 cardiometabolic traits in a large European-ancestry cohort (the UK Biobank) to their frequency in an indigenous Arab population, the MAF is on average 7.6% lower in Arabs (*p*-value = 4.2e − 06), explaining part of the reduced performance of PRS that are derived in European populations when they are applied to Arabs (Fig. 1f) [7].

The use of computational methods to improve cross-ethnic PRS performance in genetically diverse populations is encouraging, but not enough to allay real concerns regarding ongoing health disparities. While large biobanks, population cohort studies, and international consortia have allowed for serially larger GWAS, the genetic diversity of these GWAS has not kept pace, despite advocacy from groups like the American Society of Human Genetics to prioritize portability of scores. Polygenic scores are increasingly being returned to patients, and clinico-genomic models to predict and intervene on risk early in the life course through a precision medicine approach are likely to become the norm. Without the appropriate development or validation of such clinico-genomic models in Arabs, widespread implementation risks propagation of already existing disparities in care.

Beyond the serious impact on public health genomics, the massive underrepresentation of Arabs in genomic studies is a missed opportunity to discover new disease biology. A prior comment in the journal eloquently described the opportunity to identify homozygous loss of function variants and novel candidate genes for recessive disease through more sequencing of highly consanguineous Arab populations [9]. We highlight that also for common disease, homozygous loss of function variants (human knockouts) might inform interpretation of GWAS. Increasingly, the diversity of GWAS participants, more than the sample size, is advancing the discovery of new loci. For example, in a recent multi-ancestry GWAS of type 2 diabetes, 46% of new loci would not have been identified in a European-ancestry GWAS only [10]. Today, more than 30 million whole genomes have been sequenced which has advanced our understanding of genetic underpinnings of disease, but it is now well understood that in addition to the number of genomes, future opportunities for discovery lie in sequencing a diversity of genomes. First, genomes of different ancestries, when combined, allow leveraging of different LD block structures for better identification of causal variants within a specific genetic region. Second, different causal variants for similar diseases may be identified by studying diverse populations.

The unique ancestral, geographic, and cultural histories of the Arab people offer many opportunities for discovery and improved risk prediction and health for the 450 million Arabs in the world. Future directions and actions to reduce disparities and increase yield of novel variants would include (1) encouraging census bodies to disaggregate Arabs from White individuals within the diaspora, (2) active recruitment of Arabs in genomic databases, and (3) supporting infrastructure and training programs in Arab countries to develop their own biobanks genomic resources to add to the global collective pool of data.

## Data Availability

All data are publicly available at https://www.ebi.ac.uk/gwas/ and https://www.finucanelab.org/data.

## Abbreviations

BMI: Body mass index
CAD: Coronary artery disease
CB2: Cannabinoid receptor 2
DBP: Diastolic blood pressure
DKD: Diabetic kidney disease
GWAS: Genome-wide association study
HDL: High-density lipoprotein
Ht: Height
LD: Linkage disequilibrium
LDL: Low-density lipoprotein
MAF: Minor allele frequency
PC: Principal components
PRS: Polygenic risk score
QBB: Qatar Biobank
SBP: Systolic blood pressure
SNP: Single nucleotide polymorphism
T2DM: Type 2 diabetes mellitus
TG: Triglycerides
US: United States
